# Serum Profile of T Helper 1 and T Helper 2 Cytokines in Hepatitis C Virus Infected Patients

**DOI:** 10.5812/hepatmon.6156

**Published:** 2012-12-29

**Authors:** Masoomeh Sofian, Arezoo Aghakhani, Ali Asghar Farazi, Mohammad Banifazl, Ali Eslamifar, Niloofar Rashidi, Akbar Khadem Sadegh, Amitis Ramezani

**Affiliations:** 1 Tuberculosis and Pediatric Infectious Research Center (TPIRC), Arak University of Medical Sciences, Arak, IR Iran; 2 Clinical Research Department, Pasteur Institute of Iran, Tehran, IR Iran; 3 Iranian Society for Support of Patients With Infectious Diseases, Tehran, IR Iran

**Keywords:** Th1 Cells, Th2 Cells, Cytokines, Hepatitis C

## Abstract

**Background:**

T-helper (Th) lymphocyte cytokine production may be important in the immune pathogenesis of hepatitis C virus (HCV) infections. Th1 cytokines such as; interleukin-2 (IL-2), and interferon gamma (IFN-gamma) are necessary for host antiviral immune responses, while Th2 cytokines (IL-4, IL-10) can inhibit the development of these effector mechanisms.

**Objectives:**

The aim of the present study was to assess the serum profile of Th1 and Th2 cytokines in treated and non-treated HCV infected individuals.

**Patients and Methods:**

This study was carried out in 63 HCV infected patients (31 under treatment and 32 untreated) and 32 matched HCV-sero negative healthy subjects. Serum samples were checked with an enzyme-linked immune sorbent assay (ELISA) for IL-2, IL-4, IL-10 and IFN-gamma.

**Results:**

Levels of circulating IL-2, IL-4, IL-10 and IFN-gamma were significantly elevated in HCV patients versus normal controls (2 822.6 ± 1 259.92 vs. 950.8 ± 286.9 pg/mL; 1 987 ± 900.69 vs. 895.91 ± 332.33 pg/mL; 1 688.5 ± 1 405.1 vs. 519.03 ± 177.64 pg/mL and 1 501.9 ± 1 298 *vs*. 264.66 ± 71.59 pg/mL, respectively; *P* < 0.001). The serum levels of all cytokines were significantly lower in the patients under treatment than those of the untreated patients (*P* < 0.001).

**Conclusions:**

On the basis of our data, the simultaneous increase of Th1 and Th2 related cytokines may indicate that both Thl and Th2 cytokines are involved in the pathogenesis of HCV infections. Moreover, this activated T-cell response in HCV infected patients may be regulated by treatment.

## 1. Background

The hepatitis C virus (HCV) is an etiologic agent responsible for parenterally transmitted hepatitis, infecting approximately 1% of the general population worldwide (1). The clinical course of a HCV infection is highly variable, from chronic infection in a majority of cases, to self-limited infection with loss of HCV-RNA in a minority of patients (2, 3). Although the mechanism of HCV infection outcomes is not well defined, it is believed that immunological mechanisms such as cytokine production are involved in HCV pathogenesis (4, 5). Cytokines serve as the immune response molecules which have various physiological functions and regulate the immunological, inflammatory and reparative host responses, and these are mainly secreted by lymphocytes and monocytes. T cell derived cytokines are important in the host immune response.

Activated T lymphocytes are divided into two functional subsets, Thl and Th2 cells, on the basis of the cytokines that they produce (6). Thl cytokines, including interleukin-2 (IL-2) and interferon-gamma (IFN-gamma), promote a cell-mediated immunity (CMI) response whereas Th2 cytokines including IL-4 and IL-10 are involved in antibody mediated immunity. Th1 and Th2 responses have been shown to interact in a HCV infection (7, 8) and the imbalance between Th1 and Th2 responses favors humeral immune responses and down regulates cell mediated immunity, which is important for host defense against viral infections (9). Recent studies have demonstrated conflicting results on the levels of Thl and Th2 cytokines in HCV infections (10-14). While some reports have demonstrated elevated levels of IL-2, IFN-gamma (11, 15), IL-4 and IL-10 (14, 16), others have reported no increase in the levels of Thl (13, 17) and/or Th2 cytokines (15). Viral Therapy may be regulating an activated T-cell response in HCV infected patients and this creates a decreased viral load (11).

The most effective standard treatment in patients with chronic hepatitis C is a combination of pegylated interferon with ribavirin (18). The exact mechanisms by which interferon therapy alters the course of HCV disease have not been fully described. Atsukawa *et al*. (19) reported that HCV therapy polarizes the Th cell balance toward Th1 dominance and results in the reduction of Th2 cytokines, mainly IL-10. Th1 cytokines are required to eliminate HCV infected cells and impairment of these cytokines and increased levels of Th2 cytokines may be responsible for the chronicity of HCV infections (19). Therefore, the outcome of a HCV infection is related to the replication rate of the virus and the interactions between the virus and the host’s immune system (10). In addition, recent experimental studies have supported the role of immune response mechanisms in terminating HCV infections (13, 19). By further understanding the immunopathogenesis of HCV therapy, future strategies can be designed for improved HCV infection outcomes.

## 2. Objectives

The aim of the present study was to assess the serum profile of Th1 and Th2 cytokines (IL-2, IL-4, IL-10 and IFN-gamma) in treated and non-treated HCV infected individuals.

## 3. Patients and Methods

In this study, all 63 HCV infected patients who were referred to private clinics and hospitals of a central Iranian city, Arak, from January 2010 to January 2011, and 31 matched (age and sex) healthy subjects from the Arak Blood Transfusion Center, were enrolled. The 31 HCV infected patients were receiving combined pegylated interferon and ribavirin, and 32 cases did not receive any treatment. Cases and controls with the hepatitis B virus (HBV) and/or human immunodeficiency virus (HIV) infection were excluded from the study. Informed consent was obtained from all patients. A questionnaire was used to gather clinical and paraclinical data; alanine aminotransferase (ALT), viral load, and HCV genotype, and this was completed by clinicians. The project was approved by the Arak University of Medical Sciences’ Ethical Committee. Anti-HCV was tested by an enzyme-linked immune sorbent assay (ELISA) with a commercial enzyme immunoassay kit (Bio-Rad, Segrate, Italy). A recombinant immunoblot assay (RIBA Innogenetics, Ghent, Belgium) was employed to confirm anti-HCV reactivity. All subjects were tested for IL-2, IL-4, IL-10 and IFN-gamma with an ELISA (Wuhan Boster Biological Technology, Ltd., Wuhan, China). The specificity of all kits was 100%. The sensitivity of IL-2, IL-4, IL-10 and IFN-gamma kits were < 1, < 1.5, < 0.5 and < 2 pg/ml respectively. Sampling and all assay protocols, cut-offs, and result interpretations were carried out according to the manufacturers’ instructions.

### 3.1. Statistical Analysis 

The chi-square and T2 tests were calculated with the SPSS 16 package program for statistical analysis (Chicago, IL., USA). Multiple comparisons were carried out using an analysis of variance (ANOVA) test. The Spearman rank test was used for correlation. The significance level was set at *P* < 0.05. Data are presented as mean ± SD or, when indicated, as an absolute number and percentage. Unfortunately, some immunological indicators are not normally distributed, and this can minimally affect the results.

## 4. Results

A total of 63 HCV infected patients and 32 matched HCV-seronegative healthy subjects were enrolled in this study. The subjects included 31 HCV infected patients who were receiving treatment and 32 cases who did not receive any treatment. The mean age of the treated and untreated group was 33.47 ± 7.06 and 34.55 ± 8.9 years, respectively.

The possible routes of HCV transmission were injecting drug use (IDU) (60%), heterosexual contact (1.7%), IDU and infected blood (1.7%), IDU and tattooing (13.3%), heterosexual contact and intravenous drug use (3.3%), heterosexual contact and tattooing (1.7%), infected blood and tattooing (1.7%), heterosexual contact, intravenous drug use and tattooing (6.6%) and in 10% of cases the route of HCV acquisition was not identified. The mean ALT level in the HCV infected patients was 84.68 ± 180.7 IU/l and the mean log10 HCV viral load was 6.61 ± 7.10 copies/ml. The most common genotype of HCV was 3a (40%) followed by 1a (30%), 1a/b (20%), 2(6.7%) and 1b (3.3%). The levels of circulating IL-2, IL-4, IL-10, and IFN-gamma were significantly elevated in the HCV patients versus normal controls (2 822.6 ± 1 259.92 vs. 950.8 ± 286.9 pg/mL; 1 987 ± 900.69 vs. 895.91 ± 332.33 pg/mL; 1 688.5 ± 1 405.1 vs. 519.03 ± 177.64 pg/mL and 1 501.9 ± 1 298 vs. 264.66 ± 71.59 pg/mL respectively; *P* < 0.001). The serum levels of all cytokines were significantly lower in the patients under treatment than those of untreated patients (*P* < 0.001). Serum levels of IFN-gamma, IL-4, IL-10 and IL-2 in patients with chronic HCV infection and healthy controls are presented in Table 1. The results indicate that T-helper cells are activated during a HCV infection. Furthermore, the levels of IL-2 show the most dramatic elevation in patients with HCV. There was no relationship found between serum cytokines levels, HCV genotypes and possible route of HCV acquisition. The correlation between serum cytokine levels, ALT and viral load was analyzed using a Spearman’s rank test. There were no significant correlations found between cytokine levels, ALT and viral load.

**Table 1:** Comparison of Serum Cytokines in the Three Groups

	IFN-gamma (pg/ml)	IL-2 (pg/ml)	IL-4 (pg/ml)	IL-10 (pg/ml)	*P* value
HCV treated patients, Mean ± SD	784.81 ± 679.49	2026.9 ± 706.84	1512 ± 321.06	884.45 ± 1119.93	< 0.001
HCV untreated patients, Mean ± SD	2196.5 ± 1382.21	3593.4 ± 1201.98	2447.2 ± 1038.5	2467.5 ± 1209.12	< 0.001
Controls, Mean ± SD	264.66 ± 71.59	950.81 ± 286.94	895.91 ± 332.33	519.03 ± 177.64	< 0.001

Abbreviation: IFN, interferon; IL, interleukin; HCV, hepatitis C virus

## 5. Discussion

In this study, the levels of Th1 and Th2 cytokines (IL-2, IL-4, IL-10 and IFN-gamma) were assessed in HCV infected individuals. Our survey showed that the average serum levels of these cytokines were significantly higher in HCV infected patients than in those of the controls and they were also significantly higher in patients who received treatment than in the untreated HCV infected patients. Our study did not demonstrate a Th1 to Th2 shift in HCV infected patients. T-helper lymphocyte cytokine production may be important in the immune pathogenesis of HCV infections. Th1 cytokines are necessary for host antiviral immune responses, while Th2 cytokines can inhibit the development of these effector mechanisms (20). Many studies have been conducted on the importance of Thl/Th2 cytokine profiles in chronic HCV infections (11-13, 15, 17, 21). There are conflicting data from these studies regarding the levels of Th1/ Th2 cytokines in a HCV infection. Although in some surveys serum levels of Thl cytokines, including IFN-gamma and IL-2 have been reported to be elevated in HCV infections (11), some others have shown low levels of IFN-gamma in patients with HCV infections (10). Napoli *et al*. (22) found that IFN-gamma and IL-2 mRNA were increased in the livers of patients with chronic HCV. They suggested that the role of Thl cytokines is in mediating hepatocellular damage. Osna *et al*. (13) showed lower IFN-gamma and higher IL-10 levels in chronic HCV patients, than in healthy controls. Abayli *et al*. (10) also revealed an enhanced Th2 response during chronic HCV infections. A study by Chen *et al*. (1) reported that serum levels of IL-4 and IL-10 were significantly higher in HCV patients than in the controls. Another survey by Fan *et al*. (12) showed that IL-2, IL-4 and IL-10 levels were significantly increased in HCV infected hosts when compared to normal controls, but the production of Th2 cytokines was more predominant. Reiser *et al*. (21) demonstrated elevated serum IL-4 and IL-10 levels in patients with chronic HCV infection. Cacciarelli *et al*. (11) showed that levels of circulating IL-2, IL-4, IL-10, and IFN-gamma were significantly elevated in HCV patients versus normal controls and that treatment with IFN-alpha decreased the levels of IL-4, and IL-10. Another study also showed that the levels of Th2 cytokines (IL-4 and IL-10) were significantly increased in chronic HCV infected patients, compared with normal controls (23). The other investigation reported that cytokine levels in HCV patients were similar to levels observed in healthy volunteers. During IFN-alpha and ribavirin therapy no statistically significant changes in cytokine levels were observed in patients who achieved a sustained virological response, compared to unsuccessfully treated patients (24). The discrepancy between these studies may be due to epidemiological and geographic variations such as; small sample sizes, ethnic differences, comorbid conditions and composition of the study populations. Our survey showed elevated levels of Th1 and Th2 cytokines among HCV infected individuals. We did not find a Th1 to Th2 shift in these patients. Our results are in agreement with studies by Cacciarelli *et al*. (11) and Fan *et al*. (12). In the present study, decreased cytokine levels were demonstrated in patients under treatment. Cacciarelli *et al*. (11) also showed a trend toward decreased levels of cytokines during therapy. The limitations of our study are the small sample size and conducting a cross-sectional study instead of a longitudinal study. We also acknowledge the lack of detailed clinical histories and pathological records as a limitation. In conclusion, on the basis of our data, the simultaneous increase of Th1 and Th2 related cytokines may indicate that both Thl and Th2 cytokines are involved in the pathogenesis of HCV infections. In addition, this activated T-cell response in HCV infected patients could be regulated by treatment. Our data provides some additional evidence for the involvement of an immune cellular immune response in terminating HCV infections. However, further studies, including longitudinal studies as well as a larger population sample, are necessary to confirm our findings.
